# Traits determine dispersal and colonization abilities of microbes

**DOI:** 10.1128/aem.02055-24

**Published:** 2025-02-20

**Authors:** Isidora Echenique-Subiabre, Sara L. Jackrel, Jay McCarren, Chase C. James, Elisabet Perez-Coronel, Cindy Tran, Madeline Perreault, Ugbad Farah, P. Signe White, Henry K. Baker, Christopher B. Wall, Lindsay Sager, Scott Becker, Andrew D. Barton, Jonathan B. Shurin

**Affiliations:** 1 Department of Ecology, Behavior & Evolution, University of California San Diego 8784 , La Jolla, California, USA; 2 Viridos, Inc., La Jolla, California, USA; 3 Scripps Institution of Oceanography, University of California San Diego 70015 , La Jolla, California, USA; Norwegian University of Life Sciences, Ås, Norway

**Keywords:** aerosols, airborne, community assembly, trait tradeoff

## Abstract

Many microbes disperse through the air, yet the phenotypic traits that enhance or constrain aerial dispersal or allow successful colonization of new habitats are poorly understood. We used a metabarcoding bacterial and eukaryotic data set to explore the trait structures of the aquatic, terrestrial, and airborne microbial communities near the Salton Sea, California, as well as those colonizing a series of experimental aquatic mesocosms. We assigned taxonomic identities to amplicon sequence variants (ASVs) and matched them to functional trait values through published papers and databases that infer phenotypic and/or metabolic traits information from taxonomy. We asked what traits distinguish successful microbial dispersers and/or colonizers from terrestrial and aquatic source communities. Our study found broad differences in taxonomic and trait composition between dispersers and colonizers compared to the source soil and water communities. Dispersers were characterized by larger cell diameters, colony formation, and fermentation abilities, while colonizers tended to be phototrophs that form mucilage and have siliceous coverings. Shorter population doubling times, spore-, and/or cyst-forming organisms were more abundant among the dispersers and colonizers than the sources. These results show that the capacity for aerial dispersal and colonization varies among microbial functional groups and taxa and is related to traits that affect other functions like resource acquisition, predator avoidance, and reproduction. The ability to disperse and colonize new habitats may therefore distinguish microbial guilds based on tradeoffs among alternate ecological strategies.

**IMPORTANCE:**

Microbes have long been thought to disperse rapidly across biogeographic barriers; however, whether dispersal or colonization vary among taxa or groups or is related to cellular traits remains unknown. We use a novel approach to understand how microorganisms disperse and establish themselves in different environments by looking at their traits (physiology, morphology, life history, and behavior characteristics). By collecting samples from habitats including water, soil, and the air and colonizing experimental tanks, we found dispersal and invasion vary among microorganisms. Some taxa and functional groups are found more often in the air or colonizing aquatic environments, while others that are commonly found in the soil or water rarely disperse or invade new habitat. Interestingly, the traits that help microorganisms survive and thrive also play a role in their ability to disperse and colonize. These findings have significant implications for understanding microorganisms’ success and adaptation to new environments.

## INTRODUCTION

“Everything is everywhere, but, the environment selects” is a tenet of microbiology proposed by Baas-Becking in 1934 (1) that implies that dispersal places minimal constraints on the biogeography and diversity of microbes. The cosmopolitan distributions of many taxa, with the same organisms found across comparable environments anywhere on Earth, further supports the idea that microbes disperse widely and often (2). Microbes can disperse distance up to 2,000 km in the troposphere (3). Despite this high capacity for dispersal, many of the biogeographical patterns that apply to animals and plants such as the distance decay (4, 5), latitudinal diversity gradient (6), and the species-area relationship (7) have also been observed in microbial communities. However, these relationships are environment and scale dependent and may be quantitatively or qualitatively different from those shown by macro-organisms (8, 9).

For higher organisms such as plants and metazoans, several traits that affect dispersal have been identified, including seed characteristics of plants and body size for animals (10, 11). However, the traits that control rates of movements among microbial taxa are unknown. The aerial microbial communities are diverse and distinct in taxonomic composition from other environments, suggesting that some groups are more prone to airborne dispersal than others (12, 13). The presence of dormant stages, cell motility, size, or other features may influence the ability of microbes to become airborne, survive during dispersal, move among environments, and colonize new habitats (14). Whether dispersal ability is associated with other biochemical, morphological, life history, trophic strategies, or ecological traits remains unknown.

Tools have been developed to quantify and infer microbial traits based on the prediction of functional traits from taxonomic assignment of 16S and 18S rRNA gene sequences (15). These trait assignments offer an effective characterization of functional community profiling based on the most commonly used genomic regions for taxonomy. Microbial trait databases inferred from taxonomy have proven useful in the understanding of community assembly and functional structure in previous studies (15), for example, in bacteria inhabiting soil (16, 17), leaf decomposers (18), archaeal and bacterial communities from marine, fresh waters, sediments, host-associated and thermal habitats (19–21), marine mixotrophs (22), coastal protists (23), and human guts (24) among others.

We studied the taxonomic and trait composition of eukaryotic and bacterial communities from a data set that originated from an industrial algal production facility for monitoring purposes. The study was designed to detect the spread of cultivated algae in natural environments and the colonization of production ponds by wild taxa. The sampling area is in the Imperial Valley (California, USA) near the Salton Sea and included 1,054 samples collected over a period of 3 years. We analyzed this data set to test the hypothesis that trait distributions distinguish the aerially dispersing and aquatic colonizer communities from the terrestrial and aquatic sources and to ask if microbial taxa or functional traits vary in dispersal or colonization abilities.

We sampled microbes from (i) natural aquatic and (ii) terrestrial source environments, as well as (iii) aerosol samples capturing the transfer phase of dispersal, and (iv) experimental aquatic mesocosms filled with phytoplankton growth media capturing the colonization phase of aquatic organisms. Dispersal and colonization represent distinct phases of movement among habitats (25), and different traits may affect the ability of microorganisms to move through the atmosphere vs colonize new habitat. The presence of a microbe in the air indicates its potential for dispersal as airborne microorganisms may be in transit between habitats but may not become successfully established. We compared community and trait composition based on 16S and 18S rRNA gene sequences from the microbial communities present in these four sample types. We assigned taxonomic identities to ASVs and then matched these ASVs to trait values through an extensive search of published papers and reference trait databases focusing on phenotypic and metabolic traits. For 20,994 bacterial ASVs, we categorized 23 phenotypic and 92 metabolic traits using Madin et al. (21) and FAPROTAX (19), respectively. For eukaryotic ASVs following the approach of Ramond et al. (23), we categorized 10 phenotypic traits for 3,129 ASVs using HELCOM (26) and PHYLOPARS (27) databases together with specialized papers in the primary literature. If taxa or functional traits of bacterial or eukaryotic microbes vary in dispersal or colonization capacity in our data set, we expected to find differences in the frequency of different taxa or traits between the source, dispersing, and colonizing communities. Traits that occurred more often among dispersers or colonizers are hypothesized to favor movement and/or invasion. By contrast, traits that are common among the sources but rare in the dispersers and colonizers may inhibit aerial spread or rapid invasion of new aquatic habitats.

## MATERIALS AND METHODS

### Sampling sites and data collection

**Fig 1:**
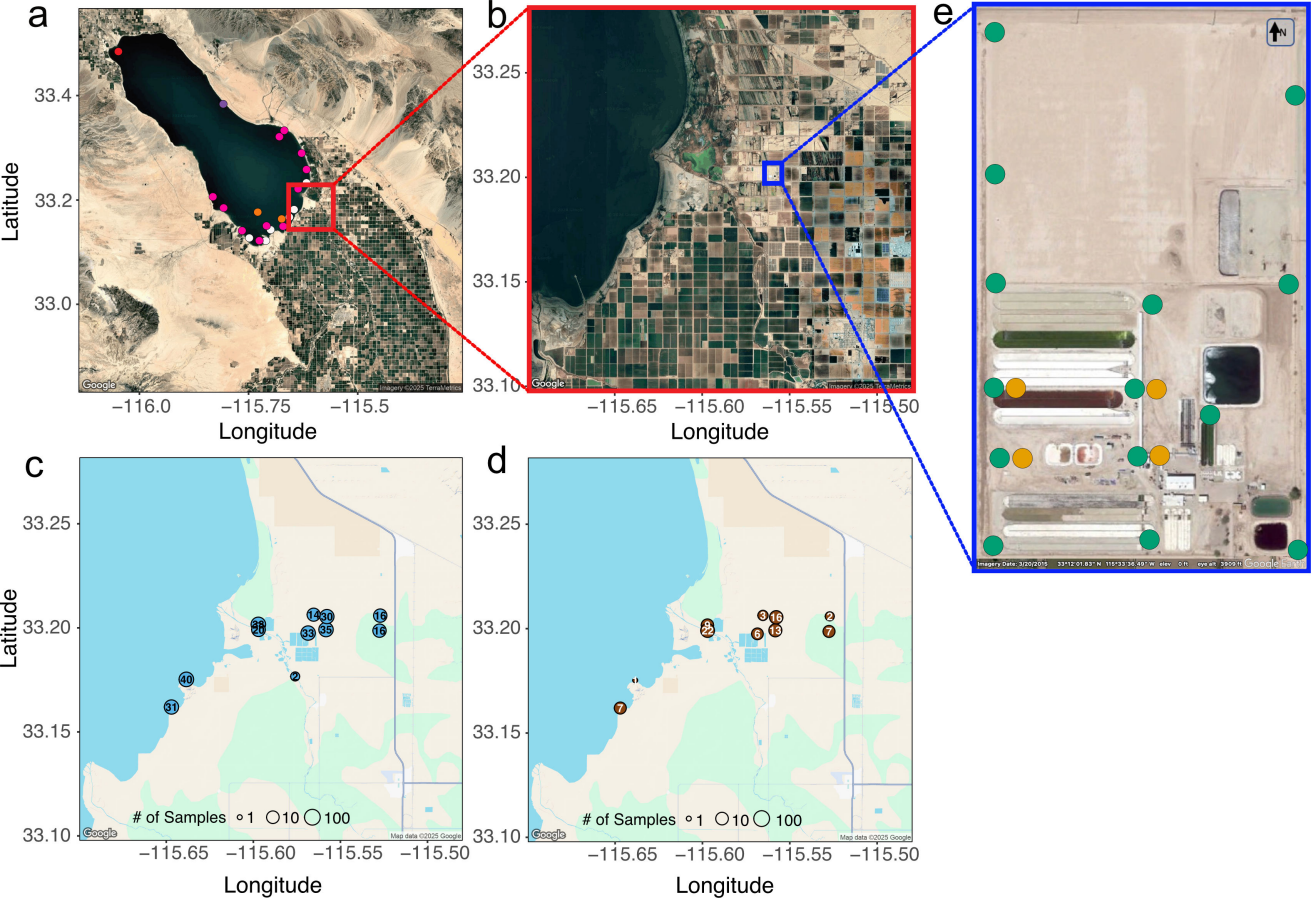
Geographic location and characterization of sampling sites. (**a**) General overview of the geographic location, circles represent detailed spatial location of samples collected from the Salton Sea. Sampling dates are indicated by color: white = 15 January 2019 (*n* = 6), orange = 30 January 2019 (*n* = 2), pink = 17 May 2019 (*n* = 11), red = 27 June 2019 (*n* = 1), and purple = 10 July 2019 (*n* = 1). Inset (red rectangle) in (**a**) shows the location of the sampling area in (**b**) where (**c**) Environmental water samples in light blue circles and (**d**) Soil samples in brown circles were collected at each environment. Inset (blue rectangle) in (**b**) shows the sampling area inside Viridos facility in (**e**) where aerosol (in orange) and open colonizing tanks (in green) samples were collected. Open colonizing tanks were located in concentric rings 100 m, 200 m, 365 m, and 550 m apart. Geographic maps in (**a–d**) were obtained from Google Maps and (**e**) was obtained from Google Earth Pro.

The data set from this study was collected to monitor the escape of cultivated algae inside and outside its industrial production facility owned by Viridos, Inc. (GPS coordinates 33.199520, –115.559237; Imperial Valley, California, USA); this included the use of air traps, colonizing tanks, and the collection of soil and water samples in the surrounding areas (Fig. 1). In total, 1,054 samples were collected between 2018 and 2020, and we characterized the four microbial communities originated from the four main studied environments: aquatic and terrestrial source environments, aerial dispersers, and tank colonizers. To characterize the aquatic and terrestrial source environments, we collected samples from water bodies (Fig. 1a and c; *n* = 275) and surrounding soil (Fig. 1d; *n* = 86) in and around the Salton Sea, California, including Salton Sea Beach, Morton Bay, marshes, rivers, and artificial ponds. We collected samples monthly from 11 specified sites from February 2018 through December 2020 on foot along the shoreline. An additional 21 samples were collected opportunistically from various locations on the Salton Sea itself when the opportunity for sampling by boat arose (Fig. 1a). Soil samples were collected from the surface in sterile sampling tubes. The water and soil samples represented the microbial source communities in the background microbial assemblage of the study area.

For colonizing tanks and aerial dispersers community sample collection, we placed open experimental tanks (Fig. 1e; in green *n* = 615) and aerosol traps (Fig. 1e; in yellow *n* = 78) at the main sampling site (Fig. 1b and e), and samples were collected in several phases corresponding to monitoring operations. Phase 1 spanned from 21 June 2019 through 26 July 2019 and contained 70 experimental tank samples and nine aerosol samples. Phase 2 spanned 10 September 2019 through 29 November 2019 and contained 304 experimental tank samples and 45 aerosol samples. Phase 3 spanned 21 July 2020 through 22 December 2020 and contained 241 experimental tank samples and 24 aerosol samples. Tanks were cleaned and re-started with fresh medium between phases. The open experimental tanks (14 tanks in 2019 and 10 tanks in 2020) were distributed throughout the 32.4-hectare site and filled with an artificial seawater medium 10× *f*/2 (28) for photoautotrophic algae. The open aquatic tanks were ~400L and 0.75 m^2^ surface area, allowing us to detect cells of taxa that dispersed and colonized to produce detectable DNA. The aerial dispersers were collected from aerosol traps surrounding the experimental tanks using four Bobcat samplers (Innovaprep, Drexel, MO) to capture microbes dispersed in the air.

### DNA extraction, sequencing, and preprocessing

After collection, all samples were stored on ice and processed within 24 hours. Microbial biomass from water samples were collected by vacuum filtration onto a 0.22 µm polyethersulfone membrane filter (Pall Corporation, Port Washington, NY). Depending on sample type and the amount of sediment and biomass concentration, we filtered 50–300 mL of sample to obtain sufficient biomass for subsequent analysis. Filters with collected biomass were stored in sterile tubes containing milling material at −20°C prior to genomic DNA (gDNA) extraction. We extracted DNA using the DNeasy PowerWater Kit following the manufacturer’s protocol (Qiagen, Germantown, MD). The DNA was quantified by Qubit dsDNA HS kit (Invitrogen, Waltham, MA) and normalized to a concentration of 7.0 ng/µL in nuclease-free water.

Biomass from aerosol samples was eluted off of collection filters according to the manufacturer’s recommendation using their 0.075% Tween20/Tris solution (Innovaprep, Drexel, MO) and immediately processed for DNA extraction. Soil samples were stored at −20°C prior to DNA extraction with PowerSoil Kit following the manufacturer’s protocol (Qiagen, Germantown, MD).

Normalized gDNA samples were amplified by PCR using indexed primers Uni_1046_F (5′ – WGGTGBTGCATGGYYGT – 3′) and Uni_1390_R (5′ – GACGGGCGGTGTGTACAA – 3′) which target conserved regions of both 16S and 18S SSU rRNA as described by Becker et al. (29). The resulting amplicons were pooled, and gel column purified (Macherey Nagel, Allentown, PA) prior to sequencing. Sequencing was conducted with an Illumina MiSeq employing 2 × 250 bp paired-end sequencing (Illumina, San Diego, CA) at Viridos, Inc.

We processed the sequencing data set using FASTQC (v0.11.7) (30) to verify data quality and the R package dada2 (v1.8) (31) to denoise the reads, assign counts, and classify taxonomy to ASVs. The database used to assign taxonomy was compiled by pulling all available sequences from Phytoref (32), PR2 (33), Silva-Mitochondria (34), and the RDP training set (35). ASVs were assigned through seven taxonomic levels ranging from domain to species when possible. We obtained 27,885 ASVs from the raw data set, accounting for 34,129,495 total sequences. We discarded singletons, doubletons, and any ASV with less than five counts to reduce sequencing errors. We retained 20,994 ASVs in the bacterial data set after excluding archaea, chloroplasts, mitochondria, and unclassified bacteria. For bacteria, samples were rarefied based on 5 k sequences per sample; this threshold was selected based on a tradeoff between number of sequences per sample vs sample size. For eukaryotes, we retained 3,129 ASVs that represented 4,624,675 sequences for the subsequent analysis after removing fungi, metazoan, macroalgae, plant, and unclassified eukaryotes ASVs. No rarefaction was applied to the eukaryotic data set, since this procedure risked losing all the aerosol and soil samples, which often contained low sequence numbers. We analyzed relative abundances to account for the uneven sequencing depth among sample types.

Because the aerosol traps and colonization tanks were located at a facility for production of natural products (biofuel) from algae, we excluded the two eukaryotic taxa under cultivation from our trait analysis. These occurred in the aerial and colonization tank samples and are likely over-represented due to the proximity of large culture ponds containing dense cultures. The taxa observed in the aerosol and tank samples therefore originated from source populations not in cultivation at the production site. However, excluding the two eukaryotic taxa from our analyses does not exclude the possibility that algal production ponds could be a source of other microbial populations that invaded aerial and colonizing tanks as well.

### Trait-based approach and functional characterization

Since bacterial and eukaryote trait databases are not unified and their classification of traits and modalities differ, they were analyzed separately. We assigned traits to bacterial and eukaryotic ASVs using the following approaches (Fig. S1).

#### Bacterial trait assignments

From the rarefied bacterial ASV table, two databases were used to annotate the bacterial traits, and each database was treated separately (Fig. S1a). The phenotypic trait database constructed by Madin et al. (21) included 14 phenotypic traits (e.g., cell diameter, doubling times, and sporulation), five quantitative genomic traits (e.g., genome size and GC content), and four environmental characteristics (e.g., isolation source, optimum pH, and optimum temperature for growth). Our bacterial ASV data set was assigned to phenotypic traits following the code specified by Madin et al. (21).

The second database used was the functional annotation of metabolic traits for prokaryotic taxa FAPROTAX (19). This database included metabolic traits related to the ability to carry out different chemical transformations such as nitrate respiration, methanogenesis, or fermentation among over 80 other functions. Bacterial ASVs were assigned to FAPROTAX functions following the instructions provided by creators (19).

#### Eukaryote trait assignments

Eukaryotic ASV traits were annotated based on their taxonomy (Fig. S1a) following the approach of Ramond et al. (23). We searched for biological descriptions for a total of 3,129 ASVs and annotated them with a modality for each trait. Trait information was obtained from literature sources, ranging from online databases such as HELCOM (26) and PHYLOPARS (27) to specialized papers in the primary literature. The eukaryote ASVs were assigned to:

- *Morphology traits*—(i) cell size: from pico (<2 µm), nano (2–20 µm), micro (20–200 µm), and macro (200–2000 µm) (36, 37). For the cell size classification, we used the median obtained from the maximum and minimum size range. This means that for spherical cells, we used the median cell diameter, while for cylinders, ellipses, or cones, we used the largest median value (obtained from either of the dimensions such as length, height, or width). (ii) Cell shape: nine different shapes including regular forms like sphere or cone to variable such as ameboid. (iii) Cell cover: organic, calcareous, siliceous, and naked (23); (iv) colony forming organisms: yes/no. (v) cilia presence: yes/no; (vi) flagella presence: yes/no; (vii) motility: attached, floater, gliding, and swimmer (23).
- *Physiological traits*—mucilage-forming organisms: yes/no.
- *Trophic strategy traits*—autotrophy, heterotrophy, and mixotrophy.
- *Life cycle traits*—based on the ability to form any resting stage such as spore or cyst, yes/no (see Fig. S2 for details).

The assignment of a trait to an ASV depended on how well it is described in the database or literature search and the resolution of a particular taxonomic group. For example, any ASVs identified as a Bacillaryophyta (diatom) were identified as having a siliceous cover regardless of the information on a higher level of resolution. Traits that varied within an ASV were identified as “low taxonomic resolution” if no assignment could be made with confidence at the relevant taxonomic level or if insufficient or ambiguous information was obtained.

The coverage of traits assigned to ASVs and percentage of sequences they represented in our data set (by counts) is shown in Fig. S2. The majority of ASVs were successfully assigned to a trait, and 9 out of 10 traits were assigned >50% of ASVs and abundance in sequence counts, with top assignments for cover, trophic strategy, cilia, and flagella. A very low percentage of assignments was obtained for mucilage both in terms of ASV numbers (<25%) and counts (<40%). A table compiling information on 10 binary or categorical phenotypic traits described in the literature and available in online databases was created.

Once we constructed our bacterial and eukaryote trait data sets, the sum of all the counts associated to each trait modality was calculated in each sample (Fig. S1b). That is, the total count of reads of all taxa assigned to a trait modality were summed for each individual sample. The relative abundance of each trait modality in the sample was obtained from the total number of counts per sample and the associated counts to that modality. Finally, the relative abundance of a trait modality in each sample was plotted, and differences among sample types were subsequently analyzed by analysis of variance (ANOVA). For the Madin database, the output of the traits database is obtained in abundance weighted-averages. Therefore, trait abundances are expressed as an abundance-weighted average from all the counts belonging to each sample, accounting for the trait value multiplied by its relative abundance coming from our initial input data set.

### Statistical analysis

If certain microbial traits confer advantages to overcome physical barriers for dispersal and/or establish in new habitats, we expect to see significant differences in taxonomic and/or trait structure among the aquatic and terrestrial source environments, aerial dispersers, and tank colonizers. Differences in alpha diversity (i.e., Shannon diversity) and the abundance of traits (average values) among environments were analyzed using two-way ANOVA with trait and community as independent variables, followed by multiple comparisons using Tukey’s honest significant difference (HSD) post-hoc test. To summarize and visualize dissimilarity in taxonomic and trait composition, we used a principal coordinate analysis (PCoA) based on ASV-relative abundances. We used the Bray-Curtis dissimilarities (R package *vegan*) (38) as the input matrix for the PCoA. To test for significant differences in trait and taxonomic composition among the four communities, we used a permutational multivariate analysis of variance (PERMANOVA) with the Bray-Curtis dissimilarity matrices (function “adonis” in *vegan*). The *R*^2^ statistic obtained from the PERMANOVA gives the effect size or amount of variation explained by sample type. The pseudo-F ratio (here depicted as F) compares the dissimilarities among sample type; larger F-values indicate more pronounced sample type separation, while lower F-values will indicate high overlap. A heatmap was used to illustrate FAPROTAX results. Data were transformed to relative abundance based on the read depth per sample (5,000). Relative abundance averages were calculated by each FAPROTAX function category and environmental source using the *dplyr* package (39). Differences in the mean frequency of traits (average values) among different environments were analyzed using ANOVA and Tukey’s HSD post-hoc test (R package *agricolae*) (40). All analyzes were done in R (41).

## RESULTS

### Core community analysis

**Fig 2:**
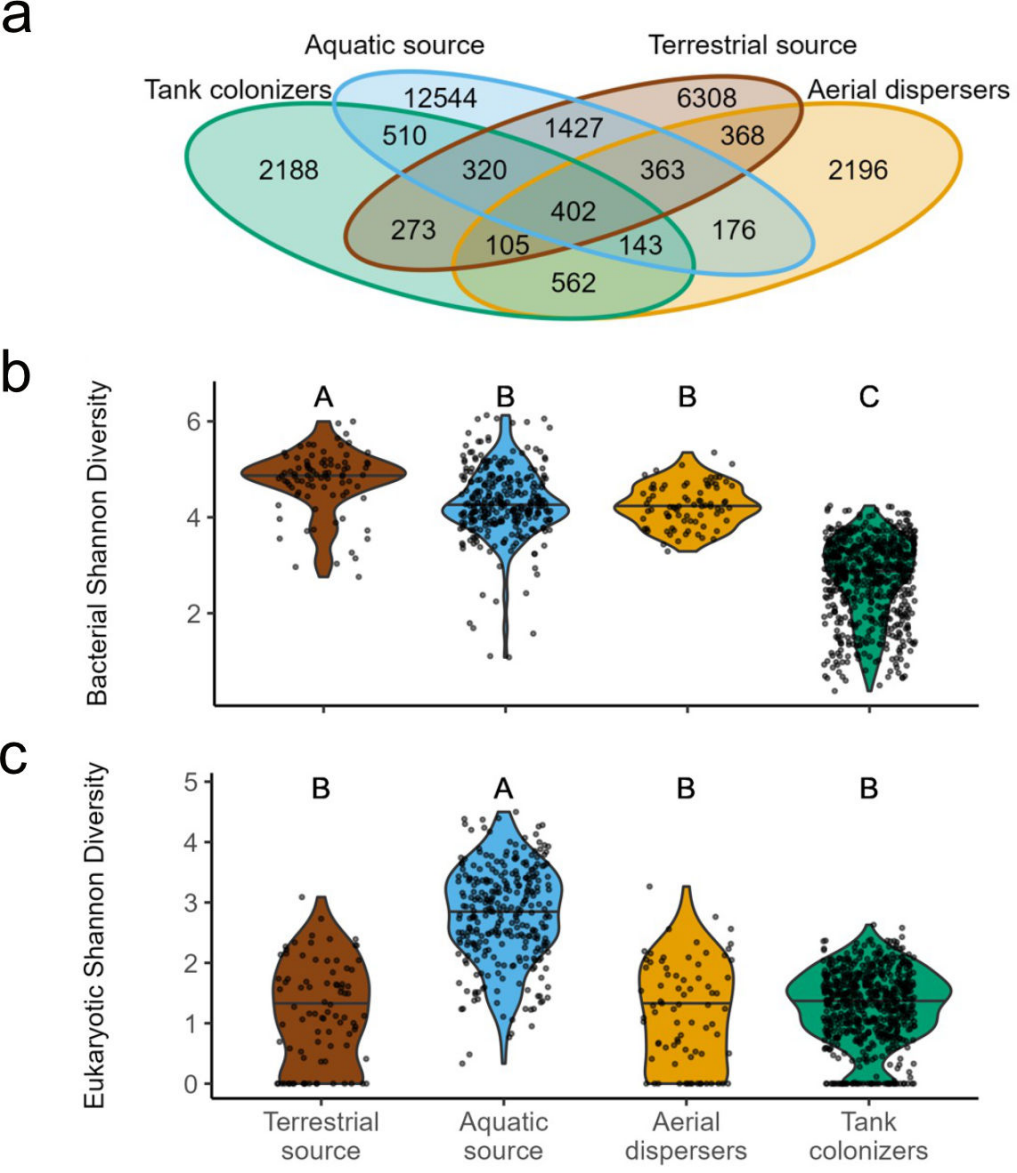
Analysis of the richness and diversity of each environment type. (**a**) Venn diagram of shared and unique ASV numbers among samples. Shannon diversity of bacterial (**b**) and eukaryotic ASVs (**c**) by environment, respectively. Letters A, B, and C in (**b**) and (**c**) denote statistically significant differences (ANOVA; Tukey’s HSD Test *P* < 0.05) in Shannon’s diversity values among the compared environments.

To characterize the alpha diversity of microbial communities from the different environments, we first focused on the total number of ASVs shared among habitats or unique to sample types. Few ASVs were shared among all communities (*n* = 402), and most were unique to only one environment (>2,000 per environment; Fig. 2a). Source environments contained more ASVs than the dispersal and colonizing communities. Shannon diversity for bacterial communities was highest in the terrestrial source community, while the tank colonizers were the least diverse (Fig. 2b; ANOVA, Tukey’s HSD, *P* < 0.002). For the eukaryotes, the aquatic source was more diverse than the rest of the environments (Fig. 2c; ANOVA, Tukey’s HSD *P* < 0.001).

### Taxonomic versus functional trait composition

**Fig 3:**
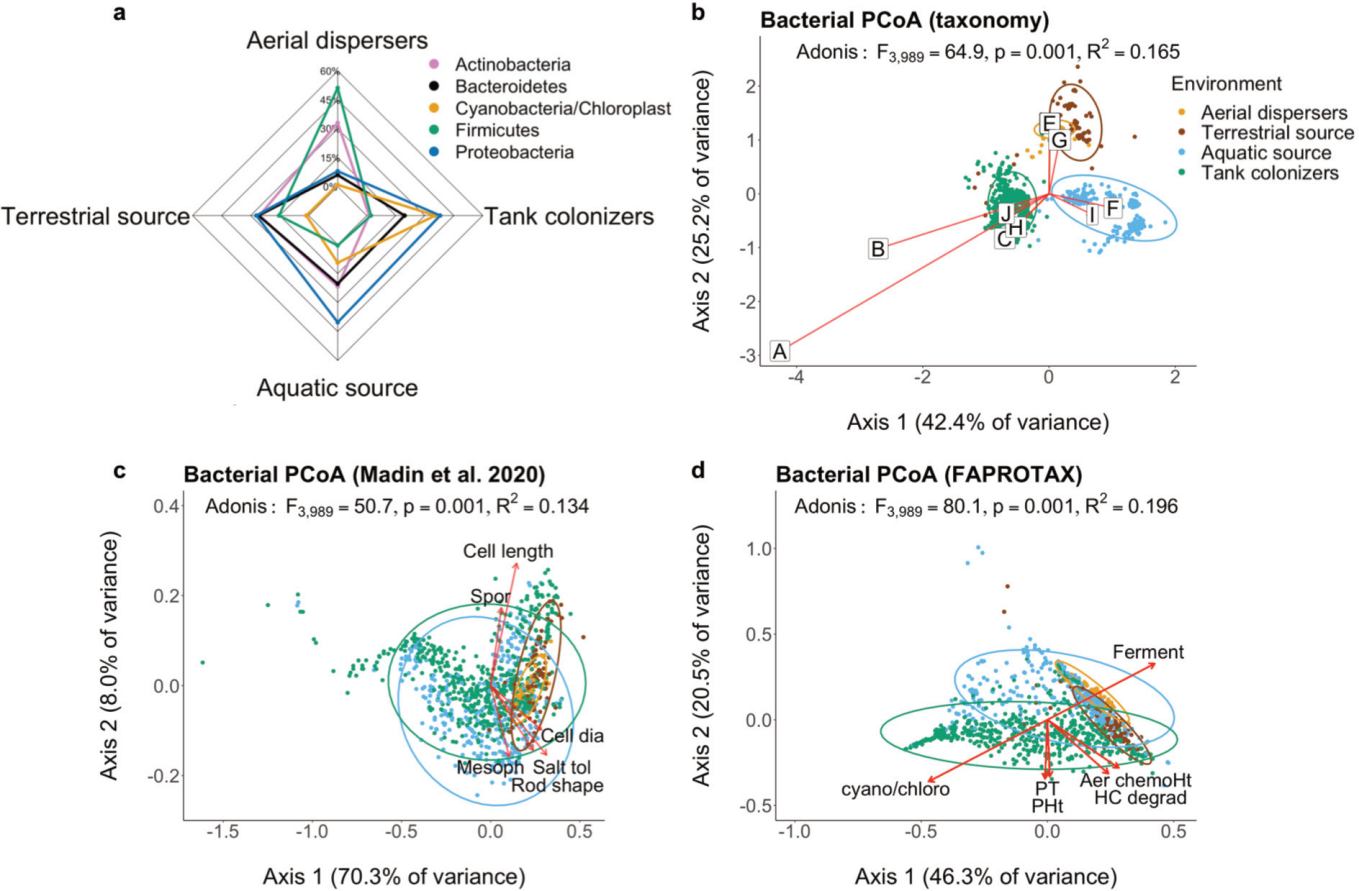
Bacterial community characterization by environment type. (**a**) Radar plots of most abundant taxonomic groups. Groups further from the center have higher relative abundances. Only taxonomic groups above 10% relative abundance are shown. Bacterial PCoA based on (**b**) taxonomic composition at the ASV level. (**c**) Madin et al. (21) phenotypic trait database and (**d**) FAPROTAX functional trait database. Arrows length for cell length in (**c**) and aerobic chemoheterotrophy and cyano/chloroplast in (**d**) were reduced by a factor of 10 to help visualize all arrows on one graph. Only the top 10 ASVs are shown on the ordination in (**b**) represented by letters: A, J: Cyanobacteria; Family IX; GpIX, B: Cyanobacteria; Family X; GpX, C: Alphaproteobacteria; Rhodobacterales; Roseivivax, D: Actinobacteria; Actinomycetales; Corynebacterium, E: Firmicutes; Bacillales; Salinicoccus, F: Betaproteobacteria; Burkholderiales; Variovorax, G: Actinobacteria; Actinomycetales; Kocuria, H: Bacteroidetes; Sphingobacteriales; Gracilimonas, I: Alphaproteobacteria; SAR11; *Candidatus* Pelagibacter. Letter D is located behind E on the plot. Results from PERMANOVA (function “adonis”) statistical test in (**b–d**) are: Pseudo F-ratio, their associated *P*-value, and the *R*^2^. Aer chemoHT, aerobic chemoheterotrophy; cyano/chloro, cyanobacteria/chloroplast; dia, diameter; Ferment, fermentation; HC degrad, hydrocarbon degradation; Mesoph, mesophilic; PHt, photoheterotrophy; PT, phototrophy; Spor, Sporulation; tol, tolerant.

**Fig 4:**
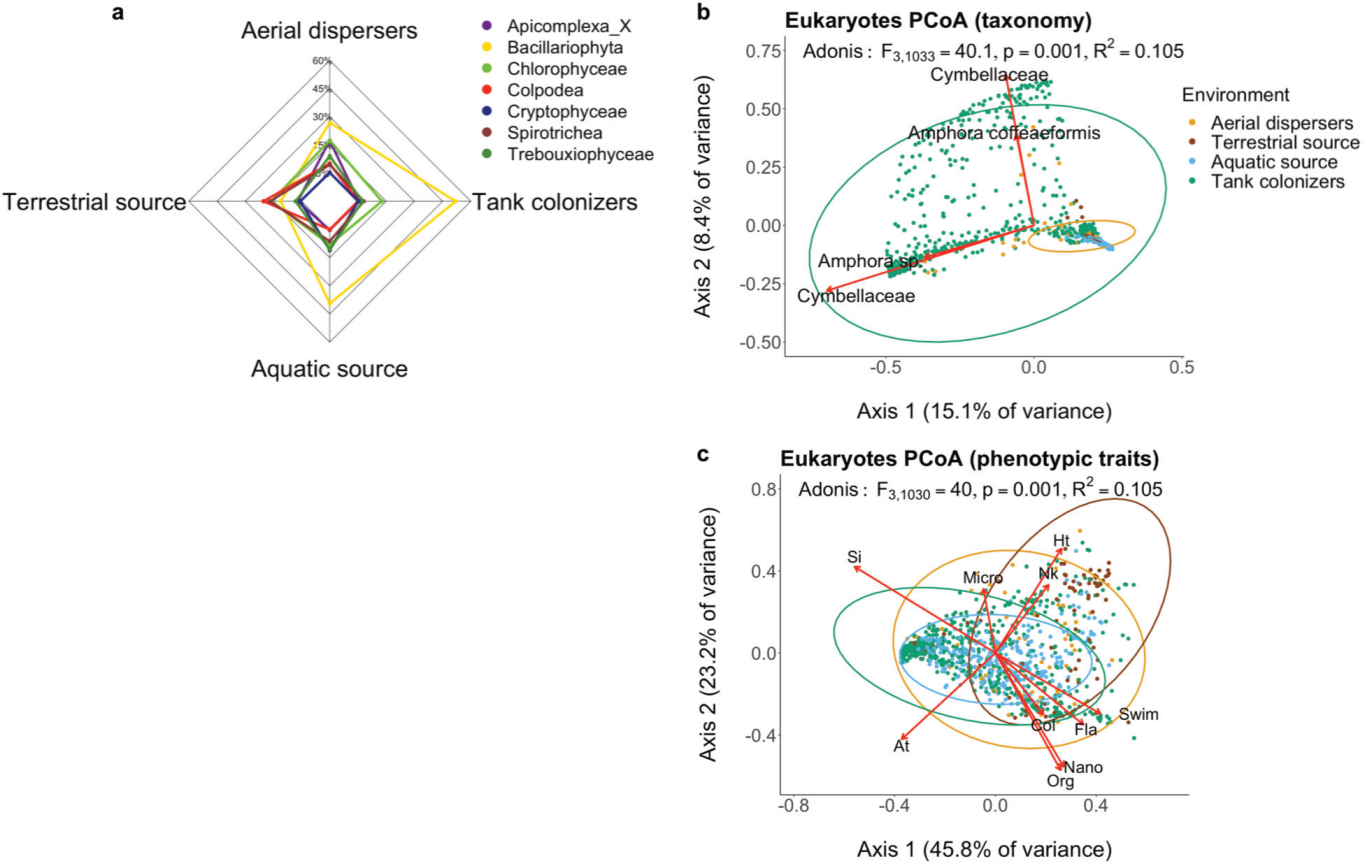
Eukaryote community characterization by environment type. (**a**) Radar plots of most abundant taxonomic groups. Groups further from the center have higher relative abundances. Only taxonomic groups above 10% relative abundance are shown. PCoA based on (**b**) taxa composition at the ASV level and (**c**) phenotypic traits. Results from PERMANOVA (function “adonis”) statistical test in (**b and c**) are Pseudo F-ratio, their associated *P*-value, and the *R*^2^. At, autotrophy; Col, colony forming; Fla, flagella; Ht, Heterotrophy; Micro, micro size; Nano, nano size; Nk, naked cover; Org, organic cover; Si, siliceous cover; Swim, swimmer.

We identified the most abundant taxonomic groups at the phylum/supergroup level among the aquatic and terrestrial source environments, aerial dispersers, and tank colonizers (Fig. 3 and 4). Only groups with >10% relative abundance in any of the community types were shown. Proteobacteria was the most abundant phylum in the aquatic source bacterial communities (~40% relative abundance; Fig. 3a). Actinobacteria, Bacteroidetes, and Proteobacteria were among the most common phyla detected in the terrestrial source (~30% relative abundance each). Actinobacteria (>30% relative abundance) and Firmicutes (>50% relative abundance) were predominant in the aerial dispersers, while Cyanobacteria and Proteobacteria were co-dominant (each >35% relative abundance) in the tank colonizers communities.

At the ASV level, the PCoA (Fig. 3b) showed strong distinction among the four bacterial communities (PERMANOVA, *F* = 64.9, *P* < 0,001). The overlap between the aerial dispersers and terrestrial sources indicates that these two groups were more similar to each other than the other communities. *Variovorax* sp. (Fig. 3b, represented by letter (F) in PCoA) and *Candidatus* Pelagibacter (I) were the dominant ASVs in the aquatic source. Aerial dispersers were dominated by *Corynebacterium* sp. (D), *Kocuria* sp. (G), and *Salinicoccus* sp. (E). Among the tank colonizers, cyanobacterial ASVs (A and B) were most abundant and identified as members of *Synechococcus* and *Cyanobacterium* genera.

The PCoA based on bacterial phenotypic traits (Fig. 3c) showed distinctions among the four communities but more overlap (PERMANOVA, *F* = 50.7, *P* < 0,001) than in the taxonomic classification (Fig. 3b, *F* = 64.9). The first axis (explaining 70.3% of total variance) separated aquatic vs terrestrial-aerial communities. Large cell diameter, salt tolerant, and rod-shaped bacteria were abundant in the terrestrial and aerial communities. The FAPROTAX-based PCoA (Fig. 3d), containing traits related to metabolic and other ecological functions, also showed a clear separation among the four communities (PERMANOVA, *F* = 80.1, *P* < 0,001). This analysis revealed that the dominant functional traits in colonizers included phototrophy, photoheterotrophy, and cyanobacterial oxygenic photoautotrophy. Aerobic chemoheterotrophy and hydrocarbon degradation were mostly prevalent in the terrestrial source, and fermentation was abundant among the aerial dispersers (Fig. S3).

Additionally, the FAPROTAX heatmap analysis (Fig. S3) revealed that another 10 functions were abundant and significantly different among the four communities; for instance, carbon and nitrogen cycling functions, such as cellulolysis, methanol oxidation, methylotrophy, nitrate reduction, nitrate respiration, and nitrogen respiration, were abundant among the aquatic sources. Aromatic compound degradation was highly abundant among terrestrial sources. Manganese oxidation, related to metal homeostasis functions, and animal parasites/symbionts were prominent among the dispersers (Fig. S3).

Among the eukaryotic communities (Fig. 4a), Bacillaryophyta (diatoms) were dominant in both the aquatic source and the tank colonizers (>30% and >45% relative abundance, respectively). Colpodea and Spirotrichea ciliates were most common in the terrestrial source (>15% compared to ≤7.5% relative abundance). Diatoms were most abundant among the aerial dispersers (~30% relative abundance); however, many other taxonomic groups, such as green algae and apicomplexans, were also detected in higher relative abundances (>15%) than the other three environments (Fig. 4a).

At the ASV level (Fig. 4b), the PCoA poorly separated the different eukaryotic communities (PERMANOVA, *F* = 40.1, *P* < 0,001) and the first two axes of the PCoA explained only 23.5% of the total variance. The dispersion of datapoints observed in the PCoA ordination for the colonizer communities revealed greater taxonomic variation than the other three communities. The diatoms *Amphora* and *Cymbellaceae* (each represented by two different ASVs) were dominant among the colonizers.

The PCoA based on eukaryotic traits (Fig. 4c) showed significant separation (PERMANOVA; *P* = 0.001) but high overlap among communities (*F* = 40). The dominant traits among the colonizers were autotrophy and siliceous cover, whereas heterotrophy and naked cells were among the common traits present in the terrestrial source (Fig. 4c).

### Univariate analysis of the trait distributions among communities

**Fig 5:**
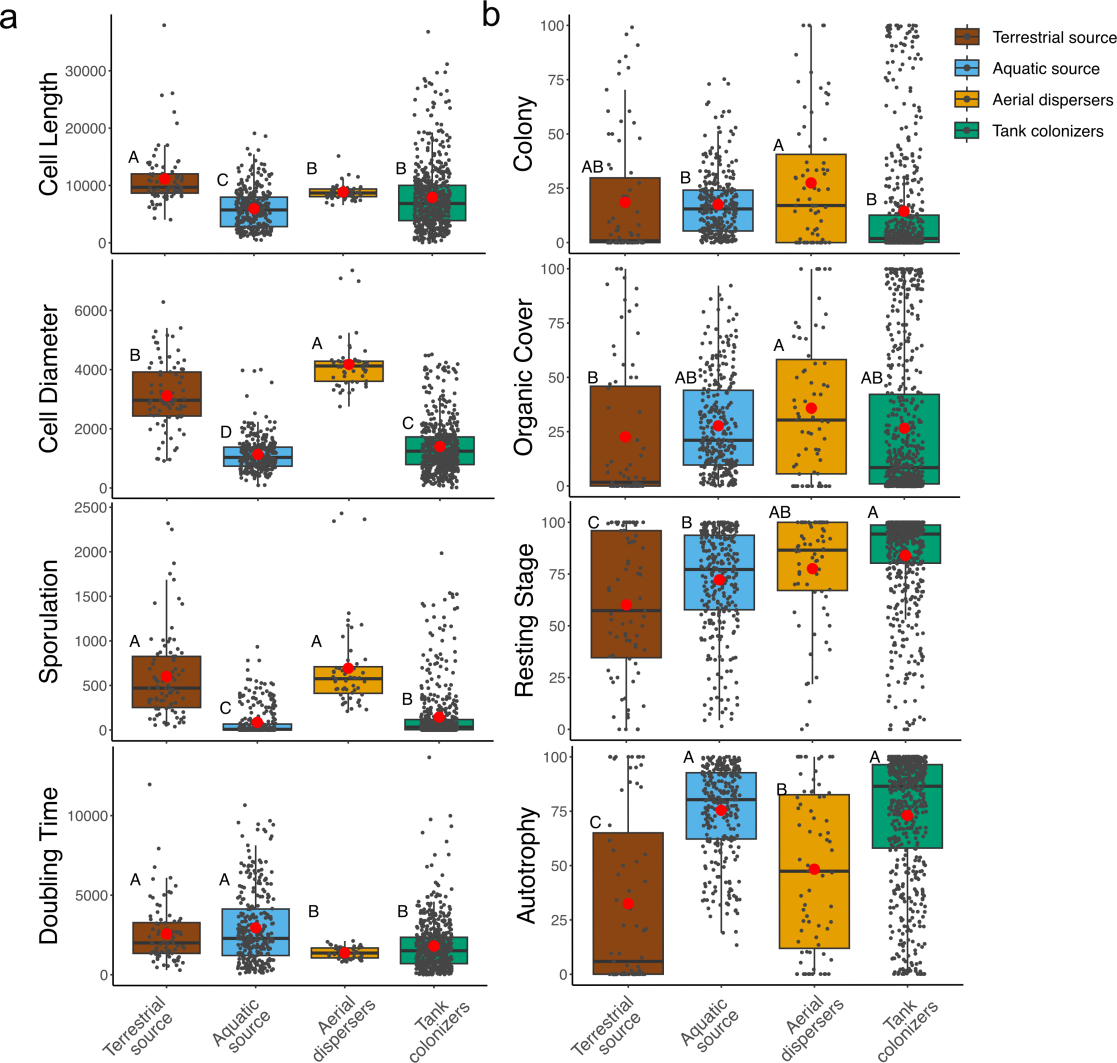
Examples of phenotypic traits that differed by environment types for (**a**) bacteria and (**b**) eukaryotes. Letters A, B, C, and D denote statistically significant differences among environments (ANOVA; Tukey’s HSD Test *P* < 0.05). For each of the microbial communities, black circles represent samples, and red dots represent the mean. In (**a**), the y axis represents abundance-weighted averages of a trait in each of the samples; this is accounting for the trait value multiplied by its relative abundance; in (**b**), the y axis represents relative abundances of that trait in each of the samples.

To characterize trait distributions among the different communities, we performed a univariate analysis of the relative abundances (abundance-weighted averages for bacteria) by an ANOVA (Fig. 5).

Among the bacterial aerial dispersers and colonizers, cell lengths were significantly lower than terrestrial sources but higher than aquatic sources (Fig. 5a). The highest average cell diameters and sporulation were found in the aerial dispersers. Doubling times were significantly lower for both aerial dispersers and colonizers compared to the source environments (Fig. 5a).

For eukaryotes (Fig. 5b), colony forming organisms were significantly more abundant among the aerial dispersers compared to both aquatic communities (source and colonizers). Organic-covered eukaryotes were most abundant among the aerial dispersers and occurred significantly more often than the terrestrial sources. Organisms with resting stages were highly abundant among the eukaryotic colonizers and aerial dispersers (Fig. 5b). Autotrophy was more abundant in aquatic communities compared to aerial dispersers and terrestrial sources, with the lowest relative abundances in the latter. No significant differences were found for abundances of flagellated cells, pico (<2 µm) or nano (2–20 µm) size eukaryotes (Fig. S4), but micro-size eukaryotes (20–200 µm) were abundant in the sources. Macro-size and mixotrophs were most abundant in the aquatic source. Terrestrial sources showed significantly higher relative abundances of naked, ciliated, swimmer, gliding, and heterotrophic eukaryotes (Fig. S4). Siliceous covered, mucilage forming, and floaters were most abundant among the tank colonizers (Fig. S4). Shape differences among communities were also commonly observed (Fig. S5). Spherical and cylindrical eukaryotes were significantly more abundant in tank colonizers.

### Colonization and dispersal abilities from an ecological perspective

**Fig 6:**
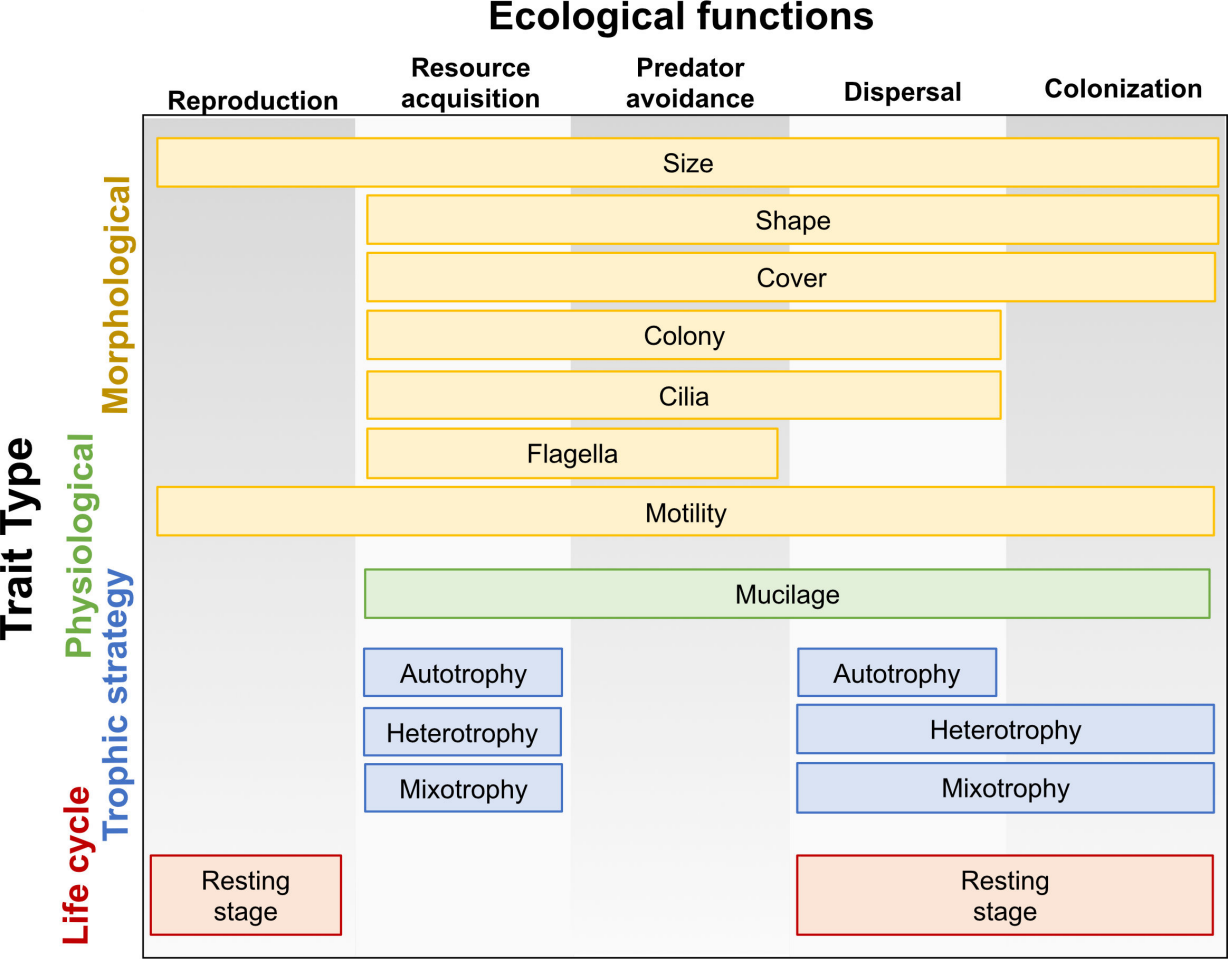
Typology of eukaryotic functional traits, modified from Litchman and Klausmeier (42) with permission.

To illustrate the traits of dispersing and colonizing eukaryotic microbes, we borrow a conceptual framework from Litchman and Klausmeier (42) linking microbial traits with different ecological functions (Fig. 6) and add categories of traits found in our study to differentiate the airborne and aquatic colonizing communities. This framework applies only to eukaryotes analyzed here. Their classification divides traits into categories related to morphology, physiology, behavior, life history, and ecological functions into reproduction, resource acquisition, and predator avoidance. We added the presence of cilia, flagella, cell cover, motility, and mucilage based on Ramond et al. (23) We included a new category called “trophic strategy” relating to energy acquisition (autotrophs, heterotrophs, and mixotrophs). According to our results, we added two new ecological functions: dispersal and colonization. We broaden this functional typology of phytoplankton functional traits to unicellular eukaryotes (protists) in general since our study includes both photosynthetic and non-photosynthetic microorganisms. Colored boxes illustrate traits in each of four categories related to the five ecological functions combining our results with Ramond et al. (23) and Litchman and Klausmeier (42) classification.

Figure 6 shows the traits related to dispersal and colonization along with the three other ecological functions identified by Litchman and Klausmeier (42). Based on our univariate analysis (Fig. 5; Fig. S4 and S5), ANOVA, and Tukey’s HSD results, traits were considered to affect colonization abilities if they were significantly over- or underrepresented in the tank colonizers pool relative to the aquatic source, while traits were considered to affect aerial dispersal ability if they were significantly over- or underrepresented in the aerial samples relative to either of the two source communities. Size, shape, cover, motility, mucilage, heterotrophy, mixotrophy, and resting stage affected both dispersal and colonization. Relative abundance of colony-forming microorganisms only differentiated aerial dispersers from the aquatic source but was equivalent between colonizers and aquatic sources. Cilia presence and autotrophy were associated with aerial dispersal, though their relative abundances in tank colonizers were equivalent to the aquatic source. There were no significant differences in relative abundances of flagellated ASVs across the community types.

## DISCUSSION

While bacterial and eukaryotic microbes from both aquatic and terrestrial environments may frequently disperse through the atmosphere, our results indicate that some microbes are better dispersers or colonizers than others, and these groups differ in their traits. Moreover, there is a lack of experimental data studying and monitoring movement of bacterial and eukaryotic microbes and the composition of the source environments. We found that microbes do not disperse equally, and their traits dictate their ability to spread and colonize new environments. We identified taxa and traits that are more prevalent among dispersers and colonizers than source environments. As expected, and consistent with previous literature, resting stages and the presence of flagella provide great advantages for dispersal abilities; however, our analysis revealed a list of new traits related to cell diameters, shape, cover, attachment, colony or mucilage forming, and trophic strategy affecting microbial dispersion and colonization.

### Differences in dispersal and colonizing abilities among taxonomic groups

Bacterial communities were more diverse than eukaryotes in all habitats and showed distinct patterns of diversity among habitats (Fig. 2b and c). Bacterial diversity decreased from sources to colonizing tanks, suggesting two potential, non-mutually exclusive scenarios: (i) many bacterial taxa that occur in soil or water rarely disperse in the atmosphere or colonize aquatic habitat and (ii) environmental filtering also plays a role by preventing their survival and establishment. The aerial dispersers also showed greatest taxonomic affinity with the soil community (Fig. 3b and 4b), consistent with previous studies showing the dispersal of airborne bacteria is facilitated by attachment to soil-dust or organic aggregates (12, 13, 43). While the Salton Sea and surrounding water bodies provide abundant sources of aquatic dispersers, the landscape surrounding our study area is primarily terrestrial (Fig. 1). Although bacteria disperse readily through the atmosphere, many taxa present in the air were absent from either the terrestrial or aquatic source environments. These taxa likely originated outside of our study area or were rare within it but frequently became airborne. The strong separation by environment observed here (Fig. 3b), indicating distinct taxonomic composition among the dispersers and colonizers, supports the idea that post-dispersal selection excludes some dispersers that are able to effectively move through the atmosphere.

The great abundance of spore-forming Actinobacteria and Firmicutes (44, 45) (relative abundances above 30%; Fig. 3a) among the dispersers provides evidence of microbes strategies for dispersal (46). Actinobacteria is a cosmopolitan and ubiquitous bacterial phylum with small cell sizes and many members that form spores. Their cell wall composition confers protection from UV-damage and resistance to desiccation, traits that likely favor aerial dispersal (45). Conversely, Actinobacteria show slow population growth rate compared to other bacterioplankton (45), suggesting that their traits allowing for dispersal may come at the cost of reduced growth within local habitats. Moreover, Actinobacteria also tend to occur more frequently in DNA-based samples than RNA-based surveys (47), suggesting that they occur broadly but are frequently inactive. High rates of dormancy could reflect a tradeoff where Actinobacteria disperse widely, but divide slowly, within different environments.

Members of the phyla Cyanobacteria, Alphaproteobacteria, and Bacteroidetes were most abundant among the aquatic colonizers, suggesting that the groups that disperse in the air are distinct from those that readily colonize aquatic habitats. Cyanobacteria were rarely found in aerial samples but abundant in the aquatic colonizing tanks, indicating that rare dispersal events may lead to successful colonization in the experimental aquatic habitats. The most abundant cyanobacteria we found in our colonizing tanks were identified as *Synechococcus elongatus*, *Cyanobacterium aponinum*, and *Synechococcus* sp. Interestingly, for the later, one of the top identity matches in BLAST was a *Synechococcus* previously collected from the Salton Sea. Cyanobacteria, particularly from freshwater habitats, are a diverse and cosmopolitan phylum of photosynthetic organisms that can rapidly proliferate (bloom) under favorable conditions (48). In fact, the *Synechococcus* genus has shown impressive plasticity and adaptability in different aquatic ecosystems, notably due to its rapid growth, stress tolerating abilities, and sophisticated strategies for light harvesting and UV protection (49). Our study is consistent with evidence showing cyanobacteria as a weedy group that successfully colonizes new habitats and achieves numerical dominance faster than other bacterial phyla even though it disperses less often. Alphaproteobacteria can degrade recalcitrant organic compounds and may be competitively dominant under low nutrient conditions or high predatory pressure (45). These bacteria are also often found in close association with eukaryotic microalgae (50). Similarly, Bacteroidetes are often found in a relationship with cyanobacteria, mainly after bloom events (45), suggesting a strong dependency on organic matter and phytoplankton blooms. The diversity of ecological strategies among the dispersing bacteria suggests that the dispersal rate as an ecological function is controlled by multiple organismal traits.

Among the eukaryotes, diatoms (Bacillariophyta) showed high dispersal and colonization abilities. Diatoms have high maximum growth rates compared with other eukaryotic phytoplankton (51) and are often efficient at storing nutrients and persisting in fluctuating resource conditions (52), allowing them to colonize and proliferate rapidly in nutrient-rich environments (53, 54). Diatoms are abundant members of the Salton Sea phytoplankton community. Their siliceous covers may protect them from grazing, making them relatively resistant to many consumers (23). Our study suggests that diatoms may also have an ecological advantage from high dispersal rates and the ability to colonize vacant habitat patches from distant sources.

### Traits affecting dispersal and colonization

#### Morphological traits

Interestingly, bacterial air dispersers had relatively larger cell diameters compared to the source habitats and colonizers (Fig. 5a). Modeling indicates that airborne microbes of ≤20 µm diameter can easily disperse and move long distances (55). Since the range of cell diameters is below this limit for bacteria analyzed in our study (56), the large cell diameters described here are still in the small size range for airborne dispersal and do not constitute a limiting factor. On the other hand, we hypothesize that smaller cell diameters, as we observed among bacterial colonizers, may be more efficient when colonizing new habitats, for instance providing an advantage over nutrient uptake due to their increasing surface-area-to-volume ratio (57). Rapid proliferation following dispersal events may explain the prevalence of small cells in the colonization tanks.

Among eukaryotes, cell size and shape vary greatly and affect microbial ecophysiological performance and fitness (51, 58, 59). Many physiological rates and ecological functions (i.e., nutrient uptake, population abundances, growth rates, and light absorption) are size and shape dependent (59, 60), having direct effects on resource acquisition and predator avoidance (42). We found that micro-size eukaryotes (20–200 µm) were more abundant in the source communities than dispersers or colonizers and did not observe significant variation among habitats for the smallest cell size classes (i.e., pico [<2 µm] and nano [2–20 µm]). This suggests the effect of size on dispersal is only apparent among larger cells (>20 µm). Shapes in microbial eukaryotes are well characterized, particularly for diatoms. We found that cylindrical and spherical shapes were abundant among the colonizers. By contrast, cell shape did not distinguish the dispersing community from the sources. Cell shape may therefore be more important for determining post-dispersal colonization than the tendency to become airborne.

Cell cover and coloniality are also distinguished among our four communities. We found colony-forming and organic-covered eukaryotes to be effective dispersers. Based on these results, we hypothesize that coloniality and organic cell cover may confer protection against desiccation and effective attachment to dust and/or organic aggregates for air dispersal (43). Moreover, coloniality might be advantageous for cells to travel further due to greater exposure to wind; however, this hypothesis needs further evaluation. Colonizers were dominated by siliceous cover—a heavy cell cover that may help resist predators (23). Among motility-associated traits, floater and attached eukaryotes were significantly more abundant among colonizers and air dispersers respectively. Cells with attaching strategies may be able to actively transport through the air attached to dust or organic particles (43). Cell size, covering, and coloniality affect resource acquisition and predator susceptibility in addition to dispersal and colonization (Fig. 6), indicating the potential for tradeoffs among these ecological functions.

#### Physiological traits

Mucilage-forming microorganisms were abundant among eukaryotic colonizers. The mucilage, a matrix of exopolysaccharides, is characteristic of phytoplankton, and its secretion can be triggered by environmental stress and nutrient deprivation (61). Mucilage is linked with drought tolerance (62) and resource acquisition since it provides a matrix for trapping particles (63) and accumulation of metal ions (61). We hypothesize that mucilage may provide protection to colonizers from environmental stress (i.e., UV, photo-inhibition, and nutrient deprivation), aid in resource capture, and provide refuge from predators. Mucilage was the most difficult trait to assign because of the lack of information available in the literature and databases (Fig. S2). Thus, we remain cautious about interpreting these results.

#### Trophic strategy

For eukaryotes, trophic strategies strongly distinguished the terrestrial and aquatic source communities, with heterotrophy most common in the soil and autotrophy in the water. The tank colonizers matched the aquatic source communities in that autotrophy was most abundant, while the aerial dispersers were more aligned with the soil communities in showing a high prevalence of heterotrophy. These patterns suggest strong selection by habitat for trophic strategies. Whether trophic strategy affects dispersal rates depends on the amount of source habitat providing propagules. The high proportion of terrestrial heterotrophs among the dispersers likely reflects the large area of terrestrial habitat relative to aquatic in our study area.

#### Life cycle traits

Microbes forming spores and resting stages were abundant in aerial dispersers for both bacteria and eukaryotes. Eukaryotes forming resting stages (i.e., spore and cyst) also occurred frequently among the tank colonizers (Fig. 5b). This is consistent with the notion that dormancy provides the ability to overcome unfavorable conditions, disperse, and successfully colonize new environments (64, 65). Dormancy is an effective strategy for dispersing in both space and time to avoid unfavorable habitat conditions and emerge when the environment improves (66). The high relative abundance of microbes with dormant life history stages among dispersers suggests that dormancy may promote both high rates of movement in the atmosphere and the ability to persist through periods of physiological stress in variable habitats.

### Revisiting Litchman and Klausmeier’s typology of phytoplankton functional traits

Our analysis is the first effort to systematically quantify how organismal traits differentiate among source, disperser, and colonizer communities of diverse bacteria and eukaryotes in a complex natural system. Figure 6 illustrates that many of the traits that distinguish eukaryotic dispersers and colonizers from aquatic and terrestrial source communities also affect reproduction, resource acquisition, and predator resistance. For instance, cell size affects all five ecological functions. Cell shape and cover, the presence of mucilage, resting stages, different trophic strategies, and forms of motility also separate dispersers and colonizers from communities in soil and aquatic habitats. The many traits that affect dispersal and colonization in addition to other ecological functions suggest that dispersal strategies of microbes have broad implications for their capacity to reproduce, compete, and resist consumers within terrestrial and aquatic habitats. Effects of traits on multiple ecological functions suggest that tradeoffs may govern community assembly and the evolution of all aspects of microbial life.

### Limitations and caveats of microbial traits studies inferred from taxonomy

Inference of microbial traits from amplicon sequencing has proven useful in the understanding of community assembly and functional structure (15, 20). However, certain limitations are inherent in this approach: (i) sequence-only approaches introduce biases due to DNA extraction, primer biases, and amplification of both live and dead cells; (ii) 16S and 18S rRNA gene sequencing errors; (iii) reliance on relative abundances instead of absolute quantification and variable sequencing depth among samples; (iv) depth and accuracy of available trait databases and appropriate references; and (v) fine-scale trait variation within an ASV (67). Despite these limitations, our work showed significant and consistent differences between taxonomy and traits composition that supported several expected patterns (e.g., more resting stages and shorter doubling times in dispersers and colonizers, more autotrophy in water, and heterotrophy in soils). Our results provide a first approach to understanding the phenotypic traits that determine microorganisms’ dispersal and colonizing abilities, and we stress the need to go beyond taxonomy inference to confirm the patterns we observed. Trait databases contain useful information about many of the traits inferred from taxonomy and are helpful for the functional characterization of microbial communities. With advances in whole-genome sequencing, single-cell analyses, and shotgun omics analyses (such as transcriptomics, proteomics, and metabolomics), trait exploration will be refined to provide key information on the community and functional structure of microbial communities.

### Conclusions

While dispersal may not constrain the biogeography of microbes to the same degree as for macro-organisms, it plays an important role in generating patterns of microbial community composition and diversity. These patterns also suggest that microbial taxa may form guilds or groups of taxa sharing similar ecological strategies due to tradeoffs between the abilities to perform vital functions linked to a common set of traits. For instance, traits that promote successful dispersal and colonization may come at a tradeoff with other ecological functions such as competition for resources or stress tolerance (68). The association of dispersal and colonization with traits known to influence other ecological functions in bacterial and eukaryotic microbes suggests that constraints on aerial dispersal may play an underappreciated role in the diversification, ecology, and biogeography of microbes. How these traits either tradeoff with or promote other fitness components in microorganisms will likely prove to be a fruitful direction for future research.

## Data Availability

Sequencing data have been archived in the NCBI Sequence Read Archive (SRA) repository under BioProject ID PRJNA1214816. Biosamples have been subsequently set up corresponding to temporally and spatially unique water, sediment/soil, aerosol, and tanks; these are BioSample IDs SAMN46404981-SAMN46405245, SAMN46406580-SAMN46406665, SAMN46406830-SAMN46406926, and SAMN46406978-SAMN46407573, respectively. The code and data files used to perform statistical analyses and generate figures for this study are available on Github (https://github.com/Isidora-hub/Flying-plankton; DOI 10.5281/zenodo.14744467).

## SUPPLEMENTAL MATERIAL

Supplemental figures aem.02055-24-s0001.docx Figures S1 to S5.

ASM does not own the copyrights to Supplemental Material that may be linked to, or accessed through, an article. The authors have granted ASM a non-exclusive, world-wide license to publish the Supplemental Material files. Please contact the corresponding author directly for reuse.

## References

[1] Baas Becking LGM. 1934. Geobiologie of inleiding tot de milieukunde. The Hague, the Netherlands.

[2] Finlay BJ. 2002. Global dispersal of free-living microbial eukaryote species. Science 296:1061–1063. doi:10.1126/science.1070710 12004115

[3] Rodó X, Pozdniakova S, Borràs S, Matsuki A, Tanimoto H, Armengol M-P, Pey I, Vila J, Muñoz L, Santamaria S, Cañas L, Morguí J-A, Fontal A, Curcoll R. 2024. Microbial richness and air chemistry in aerosols above the PBL confirm 2,000-km long-distance transport of potential human pathogens. Proc Natl Acad Sci USA 121:e2404191121. doi:10.1073/pnas.2404191121 39250672 PMC11420185

[4] Martiny JBH, Eisen JA, Penn K, Allison SD, Horner-Devine MC. 2011. Drivers of bacterial beta-diversity depend on spatial scale. Proc Natl Acad Sci USA 108:7850–7854. doi:10.1073/pnas.1016308108 21518859 PMC3093525

[5] Villarino E, Watson JR, Chust G, Woodill AJ, Klempay B, Jonsson B, Gasol JM, Logares R, Massana R, Giner CR, Salazar G, Alvarez‐Salgado XA, Catala TS, Duarte CM, Agusti S, Mauro F, Irigoien X, Barton AD. 2022. Global beta diversity patterns of microbial communities in the surface and deep ocean. Global Ecol Biogeogr 31:2323–2336. doi:10.1111/geb.13572

[6] Ibarbalz FM, Henry N, Brandão MC, Martini S, Busseni G, Byrne H, Coelho LP, Endo H, Gasol JM, Gregory AC, et al.. 2019. Global trends in marine plankton diversity across kingdoms of life. Cell 179:1084–1097. doi:10.1016/j.cell.2019.10.008 31730851 PMC6912166

[7] Horner-Devine MC, Lage M, Hughes JB, Bohannan BJM. 2004. A taxa-area relationship for bacteria. Nature 432:750–753. doi:10.1038/nature03073 15592412

[8] Astorga A, Oksanen J, Luoto M, Soininen J, Virtanen R, Muotka T. 2012. Distance decay of similarity in freshwater communities: do macro- and microorganisms follow the same rules? Glob Ecol Biogeogr 21:365–375. doi:10.1111/j.1466-8238.2011.00681.x

[9] Fierer N, McCain CM, Meir P, Zimmermann M, Rapp JM, Silman MR, Knight R. 2011. Microbes do not follow the elevational diversity patterns of plants and animals. Ecology 92:797–804. doi:10.1890/10-1170.1 21661542

[10] Leishman MR, Wright IJ, Moles AT, Westoby M. 2000. The evolutionary ecology of seed size, p 31–57. In Seeds: the ecology of regeneration in plant communities. CABI Publishing, UK.

[11] Shurin JB, Cottenie K, Hillebrand H. 2009. Spatial autocorrelation and dispersal limitation in freshwater organisms. Oecologia 159:151–159. doi:10.1007/s00442-008-1174-z 18941791

[12] Zhao J, Jin L, Wu D, Xie J-W, Li J, Fu X-W, Cong Z-Y, Fu P-Q, Zhang Y, Luo X-S, Feng X-B, Zhang G, Tiedje JM, Li X-D. 2022. Global airborne bacterial community-interactions with Earth's microbiomes and anthropogenic activities. Proc Natl Acad Sci USA 119:e2204465119. doi:10.1073/pnas.2204465119 36215495 PMC9586312

[13] Gat D, Zimmermann R, Rudich Y. 2022. Functional genes profile of atmospheric dust in the East Mediterranean suggests widespread anthropogenic influence on aerobiome composition. JGR Biogeosciences 127:1–15. doi:10.1029/2022JG00702235251875

[14] Foissner W. 2006. Biogeography and dispersal of micro-organisms: a review emphasizing protists. Acta Protozool 45:111–136.

[15] Martini S, Larras F, Boyé A, Faure E, Aberle N, Archambault P, Bacouillard L, Beisner BE, Bittner L, Castella E, Danger M, Gauthier O, Karp‐Boss L, Lombard F, Maps F, Stemmann L, Thiébaut E, Usseglio‐Polatera P, Vogt M, Laviale M, Ayata S. 2021. Functional trait‐based approaches as a common framework for aquatic ecologists. Limnol Oceanogr 66:965–994. doi:10.1002/lno.11655

[16] Sansupa C, Wahdan SFM, Hossen S, Disayathanoowat T, Wubet T, Purahong W. 2021. Can we use functional annotation of prokaryotic taxa (FAPROTAX) to assign the ecological functions of soil bacteria? Appl Sci (Basel) 11:688. doi:10.3390/app11020688

[17] Cébron A, Zeghal E, Usseglio-Polatera P, Meyer A, Bauda P, Lemmel F, Leyval C, Maunoury-Danger F. 2021. BactoTraits – A functional trait database to evaluate how natural and man-induced changes influence the assembly of bacterial communities. Ecol Indic 130:108047. doi:10.1016/j.ecolind.2021.108047

[18] Jackrel SL, Gilbert JA, Wootton JT. 2019. The origin, succession, and predicted metabolism of bacterial communities associated with leaf decomposition. MBio 10:e01703-19. doi:10.1128/mBio.01703-19 31481384 PMC6722416

[19] Louca S, Parfrey LW, Doebeli M. 2016. Decoupling function and taxonomy in the global ocean microbiome. Science 353:1272–1277. doi:10.1126/science.aaf4507 27634532

[20] Louca S, Jacques SMS, Pires APF, Leal JS, Srivastava DS, Parfrey LW, Farjalla VF, Doebeli M. 2017. High taxonomic variability despite stable functional structure across microbial communities. Nat Ecol Evol 1:0015. doi:10.1038/s41559-016-0015 28812567

[21] Madin JS, Nielsen DA, Brbic M, Corkrey R, Danko D, Edwards K, Engqvist MKM, Fierer N, Geoghegan JL, Gillings M, et al.. 2020. A synthesis of bacterial and archaeal phenotypic trait data. Sci Data 7:170. doi:10.1038/s41597-020-0497-4 32503990 PMC7275036

[22] Faure E, Not F, Benoiston A-S, Labadie K, Bittner L, Ayata S-D. 2019. Mixotrophic protists display contrasted biogeographies in the global ocean. ISME J 13:1072–1083. doi:10.1038/s41396-018-0340-5 30643201 PMC6461780

[23] Ramond P, Sourisseau M, Simon N, Romac S, Schmitt S, Rigaut-Jalabert F, Henry N, de Vargas C, Siano R. 2019. Coupling between taxonomic and functional diversity in protistan coastal communities. Environ Microbiol 21:730–749. doi:10.1111/1462-2920.14537 30672084

[24] Guittar J, Shade A, Litchman E. 2019. Trait-based community assembly and succession of the infant gut microbiome. Nat Commun 10:512. doi:10.1038/s41467-019-08377-w 30710083 PMC6358638

[25] Weiher E, Keddy P. 2004. Ecological assembly rules perspectives, advances, retreats. 2004th ed. Cambridge University Press.

[26] Olenina I, Hajdu S, Edler L, Wasmund N, Busch S, Göbel J, Gromisz S, Huseby S, Huttunen M, Jaanus A, Kokkonen P, Ledaine I, Niemkiewicz E. 2006. Biovolumes and size-classes of phytoplankton in the Baltic Sea. HELCOM BaltSea Environ Proc 106:144.

[27] Bruggeman J, Heringa J, Brandt BW. 2009. PhyloPars: estimation of missing parameter values using phylogeny. Nucleic Acids Res 37:W179–W184. doi:10.1093/nar/gkp370 19443453 PMC2703881

[28] Guillard RRL. 1975. Culture of phytoplankton for feeding marine invertebrates, p 29–60. In Culture of marine invertebrate animals. Springer US, Boston, MA.

[29] Becker S, Graham E, Sager L, Spreafico R, McCarren J. 2022. Universal amplicon sequencing of north imperial valley wetlands microbiomes. bioRxiv. doi:10.1101/2022.09.29.509762

[30] Andrews S, Krueger F, Segonds-Pichon A, Biggins L, Krueger C, Wingett S. 2010. FastQC: a quality control tool for high throughput sequence data. Babraham Institute. Available from: https://www.bioinformatics.babraham.ac.uk/projects/fastqc

[31] Callahan BJ, McMurdie PJ, Rosen MJ, Han AW, Johnson AJA, Holmes SP. 2016. DADA2: high-resolution sample inference from Illumina amplicon data. Nat Methods 13:581–583. doi:10.1038/nmeth.3869 27214047 PMC4927377

[32] Decelle J, Romac S, Stern RF, Bendif EM, Zingone A, Audic S, Guiry MD, Guillou L, Tessier D, Le Gall F, Gourvil P, Dos Santos AL, Probert I, Vaulot D, de Vargas C, Christen R. 2015. PhytoREF: a reference database of the plastidial 16S rRNA gene of photosynthetic eukaryotes with curated taxonomy. Mol Ecol Resour 15:1435–1445. doi:10.1111/1755-0998.12401 25740460

[33] Guillou L, Bachar D, Audic S, Bass D, Berney C, Bittner L, Boutte C, Burgaud G, de Vargas C, Decelle J, et al.. 2012. The Protist Ribosomal Reference database (PR2): a catalog of unicellular eukaryote Small Sub-Unit rRNA sequences with curated taxonomy. Nucleic Acids Res 41:D597–D604. doi:10.1093/nar/gks1160 23193267 PMC3531120

[34] Quast C, Pruesse E, Yilmaz P, Gerken J, Schweer T, Yarza P, Peplies J, Glöckner FO. 2013. The SILVA ribosomal RNA gene database project: improved data processing and web-based tools. Nucleic Acids Res 41:D590–D596. doi:10.1093/nar/gks1219 23193283 PMC3531112

[35] Cole JR, Wang Q, Fish JA, Chai B, McGarrell DM, Sun Y, Brown CT, Porras-Alfaro A, Kuske CR, Tiedje JM. 2014. Ribosomal Database Project: data and tools for high throughput rRNA analysis. Nucl Acids Res 42:D633–D642. doi:10.1093/nar/gkt1244 24288368 PMC3965039

[36] Sieburth Jm, Smetacek V, Lenz J. 1978. Pelagic ecosystem structure: heterotrophic compartments of the plankton and their relationship to plankton size fractions 1. Limnol Oceanogr 23:1256–1263. doi:10.4319/lo.1978.23.6.1256

[37] Raven JA. 1998. The twelfth tansley lecture. Small is beautiful: the picophytoplankton. Funct Ecol 12:503–513. doi:10.1046/j.1365-2435.1998.00233.x

[38] Oksanen J, Simpson GL, Blanchet FG, Solymos P, Stevens MHH, Szoecs E, Wagner H, Barbour M, Bedward M, Bolker B, et al.. 2022. vegan: community ecology package version 2.6-4

[39] Wickham H, François R, Henry L, Müller K, Vaughan D. 2023. dplyr:a grammar of data manipulation version 1.1.0

[40] Mendiburu F. 2022. Agricolae: statistical procedures for agricultural research version 1.3-5

[41] R Core Team. 2022. R: a language and environment for statistical computing. R Foundation for Statistical Computing, Vienna, Austria.

[42] Litchman E, Klausmeier CA. 2008. Trait-based community ecology of phytoplankton. Annu Rev Ecol Evol Syst 39:615–639. doi:10.1146/annurev.ecolsys.39.110707.173549

[43] Reche I, D’Orta G, Mladenov N, Winget DM, Suttle CA. 2018. Deposition rates of viruses and bacteria above the atmospheric boundary layer. ISME J 12:1154–1162. doi:10.1038/s41396-017-0042-4 29379178 PMC5864199

[44] Galperin MY. 2013. Genome diversity of spore-forming firmicutes bacterial systematics from Gram stain to 16S rRNA. Microbiol Spectr 1:1–27. doi:10.1128/microbiolspectrum.TBS-0015-2012 PMC4306282 26184964

[45] Newton RJ, Jones SE, Eiler A, McMahon KD, Bertilsson S. 2011. A guide to the natural history of freshwater lake bacteria. Microbiol Mol Biol Rev 75:14–49. doi:10.1128/MMBR.00028-10 21372319 PMC3063352

[46] Choudoir MJ, Barberán A, Menninger HL, Dunn RR, Fierer N. 2018. Variation in range size and dispersal capabilities of microbial taxa. Ecology 99:322–334. doi:10.1002/ecy.2094 29160898

[47] Jones SE, Lennon JT. 2010. Dormancy contributes to the maintenance of microbial diversity. Proc Natl Acad Sci U S A 107:5881–5886. doi:10.1073/pnas.0912765107 20231463 PMC2851880

[48] Paerl HW, Fulton RS 3rd, Moisander PH, Dyble J. 2001. Harmful freshwater algal blooms, with an emphasis on cyanobacteria. ScientificWorldJournal 1:76–113. doi:10.1100/tsw.2001.16 PMC6083932 12805693

[49] Callieri C. 2017. Synechococcus plasticity under environmental changes. FEMS Microbiol Lett 364:1–8. doi:10.1093/femsle/fnx229 29092031

[50] Jackrel SL, Yang JW, Schmidt KC, Denef VJ. 2021. Host specificity of microbiome assembly and its fitness effects in phytoplankton. ISME J 15:774–788. doi:10.1038/s41396-020-00812-x 33097853 PMC8027036

[51] Edwards KF, Thomas MK, Klausmeier CA, Litchman E. 2012. Allometric scaling and taxonomic variation in nutrient utilization traits and maximum growth rate of phytoplankton. Limnol Oceanogr 57:554–566. doi:10.4319/lo.2012.57.2.0554

[52] Marañón E. 2015. Cell size as a key determinant of phytoplankton metabolism and community structure. Ann Rev Mar Sci 7:241–264. doi:10.1146/annurev-marine-010814-015955 25062405

[53] James CC, Barton AD, Allen LZ, Lampe RH, Rabines A, Schulberg A, Zheng H, Goericke R, Goodwin KD, Allen AE. 2022. Influence of nutrient supply on plankton microbiome biodiversity and distribution in a coastal upwelling region. Nat Commun 13:2448. doi:10.1038/s41467-022-30139-4 35508497 PMC9068609

[54] Litchman E, de Tezanos Pinto P, Edwards KF, Klausmeier CA, Kremer CT, Thomas MK. 2015. Global biogeochemical impacts of phytoplankton: a trait-based perspective. J Ecol 103:1384–1396. doi:10.1111/1365-2745.12438

[55] Wilkinson DM, Koumoutsaris S, Mitchell EAD, Bey I. 2012. Modelling the effect of size on the aerial dispersal of microorganisms. J Biogeogr 39:89–97. doi:10.1111/j.1365-2699.2011.02569.x

[56] Burrows SM, Elbert W, Lawrence MG, Pöschl U. 2009. Bacteria in the global atmosphere – Part 1: review and synthesis of literature data for different ecosystems. Atmos Chem Phys 9:9263–9280. doi:10.5194/acp-9-9263-2009

[57] Levin PA, Angert ER. 2015. Small but mighty: cell size and bacteria. Cold Spring Harb Perspect Biol 7:a019216. doi:10.1101/cshperspect.a019216 26054743 PMC4484965

[58] Grover JP. 1989. Influence of cell shape and size on algal competitive ability. J Phycol 25:402–405. doi:10.1111/j.1529-8817.1989.tb00138.x

[59] Ryabov A, Kerimoglu O, Litchman E, Olenina I, Roselli L, Basset A, Stanca E, Blasius B. 2021. Shape matters: the relationship between cell geometry and diversity in phytoplankton. Ecol Lett 24:847–861. doi:10.1111/ele.13680 33471443

[60] Finkel ZV, Beardall J, Flynn KJ, Quigg A, Rees TAV, Raven JA. 2010. Phytoplankton in a changing world: cell size and elemental stoichiometry. J Plankton Res 32:119–137. doi:10.1093/plankt/fbp098

[61] Leppard GG. 1995. The characterization of algal and microbial mucilages and their aggregates in aquatic ecosystems. Sci Total Environ 165:103–131. doi:10.1016/0048-9697(95)04546-d 7754351

[62] Romaní AM, Amalfitano S, Artigas J, Fazi S, Sabater S, Timoner X, Ylla I, Zoppini A. 2013. Microbial biofilm structure and organic matter use in Mediterranean streams. Hydrobiologia 719:43–58. doi:10.1007/s10750-012-1302-y

[63] De Philippis R, Faraloni C, Sili C, Vincenzini M. 2005. Populations of exopolysaccharide-producing cyanobacteria and diatoms in the mucilaginous benthic aggregates of the Tyrrhenian Sea (Tuscan Archipelago). Sci Total Environ 353:360–368. doi:10.1016/j.scitotenv.2005.09.078 16271382

[64] Lennon JT, Jones SE. 2011. Microbial seed banks: the ecological and evolutionary implications of dormancy. Nat Rev Microbiol 9:119–130. doi:10.1038/nrmicro2504 21233850

[65] Litchman E. 2010. Invisible invaders: non-pathogenic invasive microbes in aquatic and terrestrial ecosystems. Ecol Lett 13:1560–1572. doi:10.1111/j.1461-0248.2010.01544.x 21054733

[66] Snyder RE. 2006. Multiple risk reduction mechanisms: can dormancy substitute for dispersal? Ecol Lett 9:1106–1114. doi:10.1111/j.1461-0248.2006.00962.x 16972874

[67] Baker D, Lauer J, Ortega A, Jackrel SL, Denef VJ. 2022. Effects of phycosphere bacteria on their algal host are host species-specific and not phylogenetically conserved. Microorganisms 11:62. doi:10.3390/microorganisms11010062 36677355 PMC9862884

[68] Malik AA, Martiny JBH, Brodie EL, Martiny AC, Treseder KK, Allison SD. 2020. Defining trait-based microbial strategies with consequences for soil carbon cycling under climate change. ISME J 14:1–9. doi:10.1038/s41396-019-0510-0 31554911 PMC6908601

