# Supplementary material for: Traits determine dispersal and colonization abilities of microbes
Source: Appl Environ Microbiol. 2025 Feb 20;91(3):e02055-24. doi: 10.1128/aem.02055-24 (PMC11921345; doi:10.1128/aem.02055-24)
Supplement: Supplemental figures — Figures S1 to S5. [file aem.02055-24-s0001.docx]

**Supplementary information**

**Supplementary figures**

**Figure S1**: Trait-based and workflow used in this study to infer traits from taxonomy. (a) Phenotypic databases consulted for bacterial and eukaryotic communities (b) Workflow example for categorical (trait 1) and binary (trait 2) traits assignment and quantification used in our study; **(1)** **Compile dataset**: we started by compiling the three tables of ASV counts, taxonomy and metadata, **(2)** **Assign traits based on taxonomy**: we used the taxonomy table to identify ASVs to species or the lowest resolved taxonomy possible in order to assign traits based on taxonomy, **(3)** **Combine**: once traits were assigned, we combined the traits table with the ASV counts table, **(4)** **Condense**: we combined horizontally by summing all the counts associated to each trait modality in each individual sample, **(5) Transform to relative abundances and assign metadata**: the relative abundance of each trait modality in the sample was obtained from the total number of counts per sample and the associated counts to that modality, **(6) Visualize**: the relative abundance of a trait modality in each sample was plotted and subsequently analyzed for differences among the four environments.

**Figure S2**: Completeness of eukaryotic trait data set from this study. Percentage (%) of ASVs assigned by number and by counts. Modified from Litchman and Klausmeier (42) and Ramond et al. (23). Gray boxes represent ecological functions affected by traits. By counts, we account for the total percentage of sequences assigned to a particular ASV on the whole dataset. “Assigned” refers to the ASVs that have an assigned trait; “LTR” means low taxonomic resolution; “empty” were ASVs not assigned due to no available information and/or because the ASV was not abundant on the dataset.

**
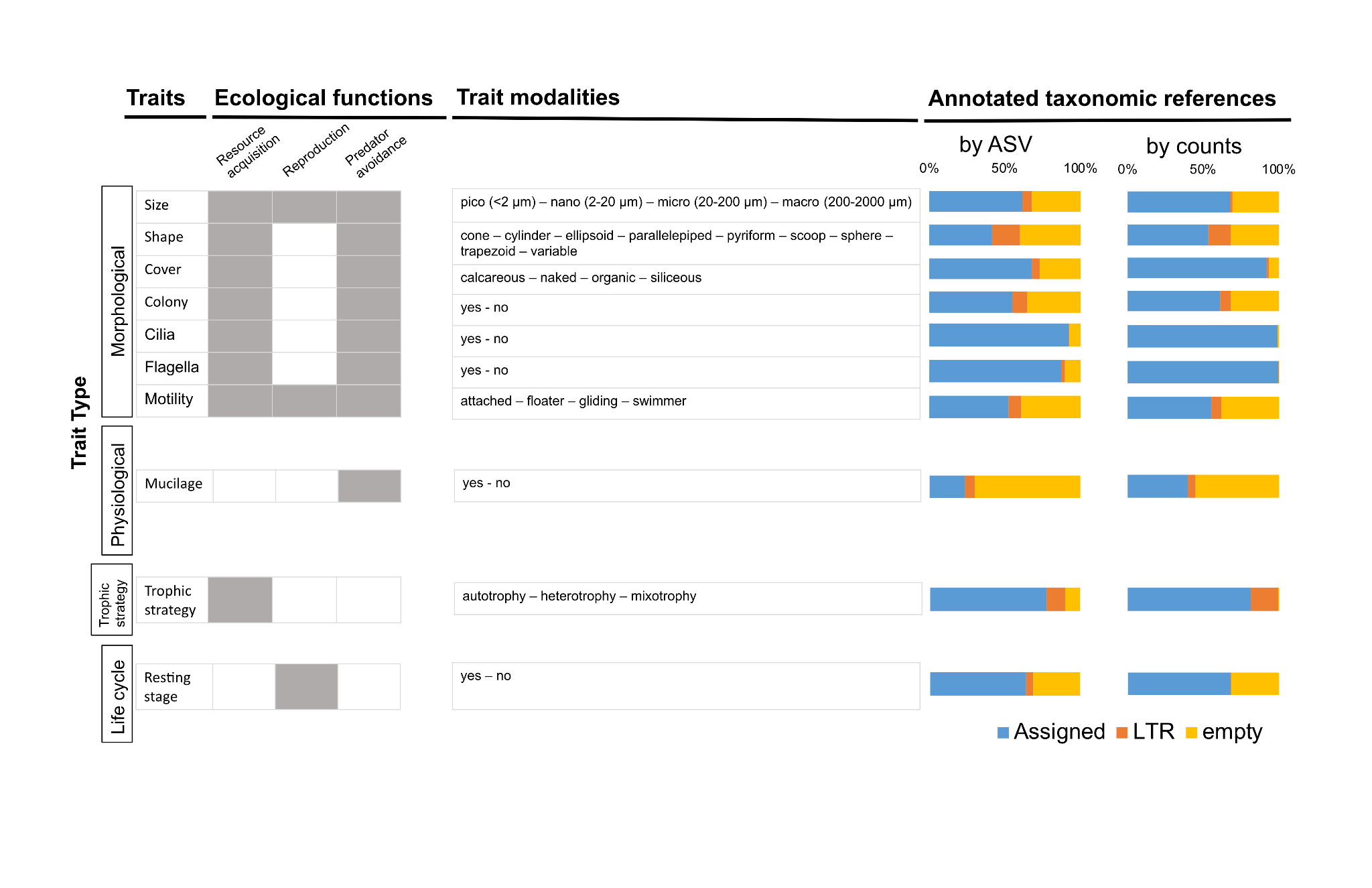
**

**Figure S3**: Bacterial FAPROTAX traits heatmap based on relative abundance per sample (blue shading). Samples were assigned to the four environments: terrestrial source, aquatic source, aerial dispersers and tank colonizers. Relative abundance averages were calculated by each FAPROTAX function category and environmental source. The abundance of traits (average values) among different environments were analyzed using ANOVA and Tukey’s HSD post-hoc test. Geometric symbols represent letters A, B, C, D to denote statistically significant differences among environments (ANOVA; Tukey’s HSD Test p < 0.05).


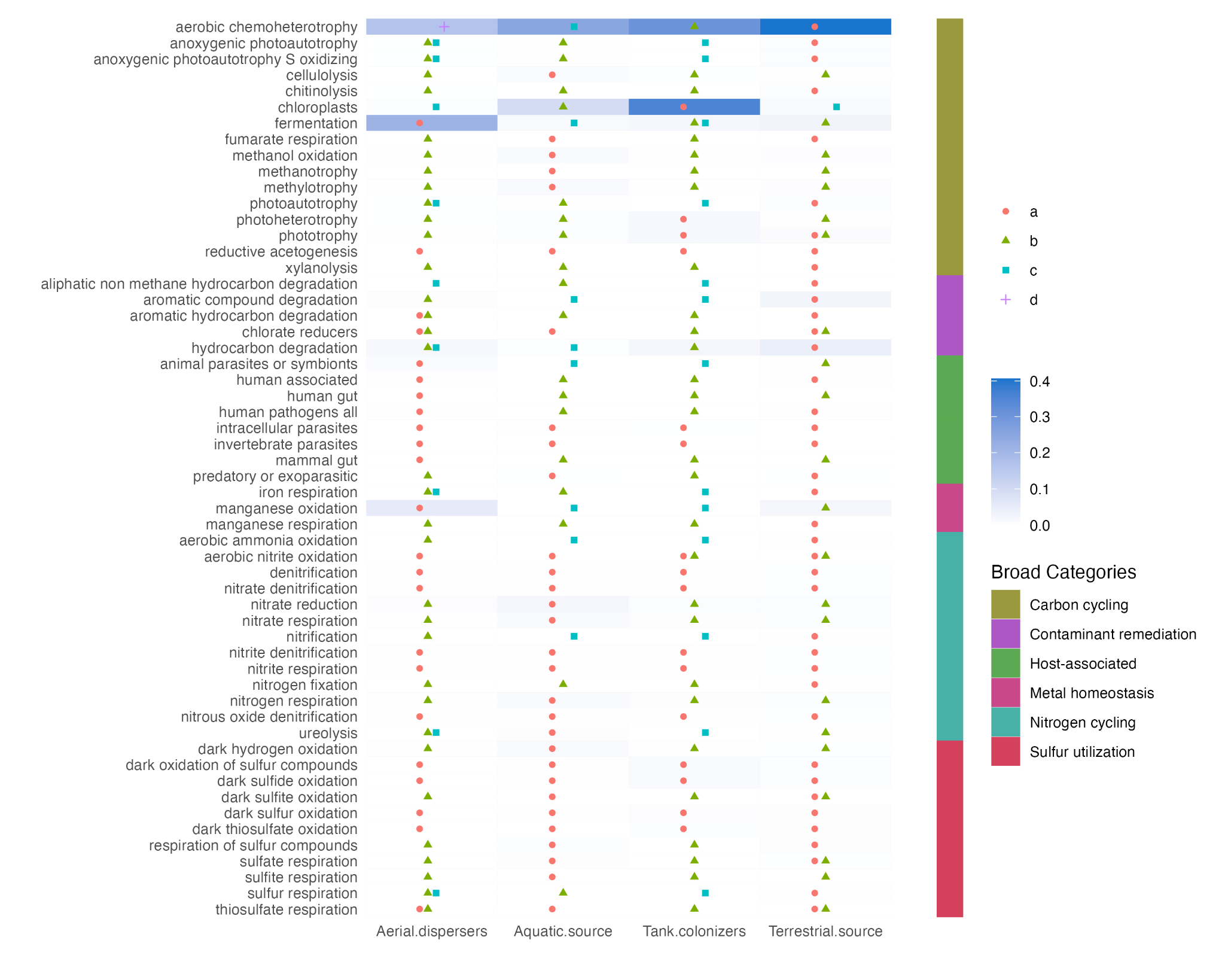


**Figure S4**: Eukaryotic traits. For each of the microbial communities, circles represent samples. The y-axis represents relative abundances of that trait in each of the samples. Letters denote statistically significant differences among environments (ANOVA; Tukey Test p < 0.05); No statistically significant differences were found among nano size modality. Note: Pico size modality is not shown here (No statistically significant differences were found and very few organisms in this data set were classified as pico sized eukaryotes).

**Figure S5**: Traits for eukaryote shapes. For each of the microbial communities, open circles represent samples. The y axis represents relative abundances of respective shapes in each of the samples. Letters denote statistically significant differences among environments (ANOVA; Tukey Test p < 0.05) at each trait modality; No statistically significant differences were found among the variable shape modalities.
